# Consecutively Preparing D-Xylose, Organosolv Lignin, and Amorphous Ultrafine Silica from Rice Husk

**DOI:** 10.1155/2014/603481

**Published:** 2014-07-22

**Authors:** Hongxi Zhang, Xuefeng Ding, Zichen Wang, Xu Zhao

**Affiliations:** ^1^Chemistry Department, Changji University, No. 77, North Beijing Road, Changji 831100, China; ^2^College of Chemistry, Jilin University, No. 2699, Qianjin Street, Changchun 130012, China

## Abstract

Rice husk is an abundant agricultural by-product reaching the output of 80 million tons annually in the world. The most common treatment method of rice husk is burning or burying, which caused serious air pollution and resource waste. In order to solve this problem, a new method is proposed to comprehensively utilize the rice husk in this paper. Firstly, the D-xylose was prepared from the semicellulose via dilute acid hydrolysis. Secondly, the lignin was separated via organic solvent pulping from the residue. Finally, the amorphous ultrafine silica was prepared via pyrolysis of the residue produced in the second process. In this way, the three main contents of rice husk (semicellulose, lignin, and silica) are consecutively converted to three fine chemicals, without solid waste produced. The yields of D-xylose and organosolv lignin reach 58.2% and 58.5%, respectively. The purity and specific surface of amorphous ultrafine silica reach 99.92% and 225.20 m^2^/g.

## 1. Introduction

Biomaterials offer the potential of renewability, biodegradation, and a path away from harmful additives. So the biomaterials hold great promise to mitigate many sustainability problems. As one of the biomaterials, rice husk (RH) is the abundant agricultural by-product in all rice producing countries. The annual output of RH of the world and China is around 80 and 40 million tons [1], respectively. Earlier reports had discussed the conversion of rice husk into some products, such as building materials [2], adsorbent [3], and biogas [4]. However, most of them are burnt openly or buried currently in China, which aroused the serious environmental pollution. Therefore, it is very urgent to find new methods to fully utilize the rice husk.

RH is composed of 18% xylan, 22% lignin, 38% cellulose, 20% amorphous silica, and 2% other organic components [5]. So the key to comprehensively utilize rice husk is the synchronous utilization of those main contents. The research reported here involves the efficient techniques for consecutively producing D-xylose, organosolv lignin, and amorphous ultrafine silica from the three main contents of rice husk.

D-Xylose is widely used in fields related to the food and pharmaceutical industries for its low calorific value and acceptable organoleptic properties. According to the research of Watanabe et al. [6], the semicellulose in RH is made up of substituted arabinoxylan, so D-xylose can be prepared via dilute acid hydrolysis [7].

Lignin is the abundant renewable aromatic biopolymer [8], which is widely used as adsorbent [9], carbon precursor for pesticide [10], active carbon [11], polymeric additive [12], and so forth. Lignin is separated from biomass via alkaline pulping (Kraft and Soda) process on the commercial scale [13]. However, this method is not suitable for separating the lignin from RH. The amorphous silica in RH can be easily transformed to the silicate and dissolved during the alkaline pulping process, which causes the loss of silica and difficulty of alkaline recycle. Organic solvent pulping is a good choice to separate lignin from RH, for it has little impact on the amorphous silica of RH. The ethanediol is chosen in this paper as the solvent for the high boiling point and good thermostability.

Ultrafine silica is widely used in electronics, ceramics, plastics, rubber, and photoelectric material industries [14]. The content of amorphous silica in RH is the highest in all Gramineae plants. Preparation of amorphous ultrafine silica from RH is the most attractive utilization method. Several reports have concluded that rice husks are an excellent source of high-grade amorphous silica [15–17]. We had reported previously that the ultrafine amorphous silica powder is obtained after the hydrolysis of semicellulose and porphyrization of RH residue [18]. However, the abundant lignin in residue is burned during the pyrolysis process at high temperature (750°C), the lignin is wasted, and the energy-consuming is high, not to mention the serious aggregation of ultrafine silica due to the high pyrolysis temperature. Therefore, how to effectively utilize the lignin in rice husk, decrease the temperature of pyrolysis, and increase the dispersity of ultrafine silica is discussed in this paper. A new method of comprehensively utilizing the semicellulose, lignin, and silica of rice husk was proposed. In this method, the semicellulose, lignin, and silica in the RH are consecutively converted to the D-xylose, organosolv lignin, and amorphous ultrafine silica in the way of carbon neutral and without producing solid waste. This method is helpful to resolve the problem of waste and pollution aroused by the rice husk.

## 2. Materials and Methods

### 2.1. Materials and Reagents

Rice husk is obtained from a rice mill nearby Changchun city, China. RH is washed thoroughly with distilled water to remove adhering soil and dust, then dried at 65°C overnight, and porphyrized to 60 meshes before employment.

All chemical reagents, such as ethanediol, sulphuric acid, and calcium oxide, are analytically pure from Sinopharm Chemical Regent Co. Ltd in China.

### 2.2. Semicellulose Hydrolysis and D-Xylose Preparation

The semicellulose in RH is hydrolyzed by dilute sulfuric acid under certain conditions. After being filtrated and washed with ethanol, the D-xylose is obtained with a purity of 98.5%. The detailed information is described previously [18].

### 2.3. Separating Lignin from Residue 1

Residue 1 referred to the residue of rice husk produced in the semicellulose hydrolysis. It is mixed with ethanediol solution at some proportion into a 50 mL stainless steel autoclave with Teflon inner lining. The autoclave is screwed up and put into an oven heated beforehand to a designed temperature and kept in a given period. After pulping is finished, the autoclave is taken out and naturally cooled to 333 K. The solid RH residue and liquid are rapidly filtrated, and the residue is washed with 333 K organic solution twice to reduce lignin deposit. The filtrate is gathered in a beaker, and triple volume of ethanediol solution distilled water is supplemented into the filtrate. The automatic precipitation of lignin takes place immediately. For the purpose of complete deposition, electromagnetic stirring is carried out. The suspension is filtrated again to separate solid lignin from spent liquid. The gained lignin is dried overnight at room temperature in the vacuum drying oven.

### 2.4. Preparation of Amorphous Ultrafine Silica from Residue 2

*The two-step method* is employed to decrease the temperature and improve the dispersity of ultrafine silica. Residue 2 referred to the RH produced in the lignin extraction process. After being dried at 65°C overnight, residue 2 is weighted and put into a tube furnace, which is heated to a certain temperature beforehand under the flowing of carbon dioxide gas (the first step); residue 2 is hydrolyzed to be black powder (a mixture of silica and carbon). Then the carbon dioxide gas flow is changed to be the oxygen flow under certain temperature (the second step). The black powder completely turns into white silica in 30 minutes.

### 2.5. Analysis

The size and morphology of silica powder are performed with a transmission electron microscope (JEOL, JEM-1200EX).

The thermal stability of the samples was performed with a thermogravimetric analyzer (Mettler Toledo, TGA/SDTA 851) in air atmospheres. Samples of about 15 mg were heated from room temperature to 900°C with the constant heating rate of 10°C/min and the gas flow rate of 20 mL/min.

The specific surface area and pore size of silica powder are measured by using the Brunauer-Emmett-Teller (BET) method with an automated chemisorption/physisorption surface area and pore size analyzer (Quantachrome AUTOSORB-1C).

The apparent weight-average molecular weight (Mw), the apparent number-average molecular weight (Mn), and the molecular weight distribution (Mw/Mn, DPI) are measured on a Gel Permeation Chromatography (WATERS 1515) instrument. The Styragel HT columns used THF as an eluent (1.0 mL/min) at 308 K. The calibration curve is obtained with linear polystyrene as standards.

Lignin is ground and mixed with KBr to make a pellet (0.5% m/m) to obtain infrared spectra with Fourier Transform Infrared spectroscopy (Shimadzu FTIR 8400).

Ultraviolet spectroscopy (UV) is carried out on a Shimadzu UV-2550 by using lignin mentioned above. Lignin and barium sulfate are mixed and pressed in the attachment of UV-2550 to get a smooth surface; then the ultraviolet adsorption spectrum of lignin is obtained by integrating sphere method.

The amorphous state of silica powder is performed on an X-ray diffractometer (Shimadzu-6000), using Cu K*α* (*λ* = 1.54056) radiation.

To measure the purity of silica powder, inductively coupled plasma (ICP) is performed on an ICP spectrometer (Agilent 7500A).

The content of the semicellulose, cellulose, lignin, and silica in the RH is measured according to the China National Standards GB/T 2677.9-94, GB/T 2677.10-95, GB/T 747-2003, and GB/T 2677.3-93.

## 3. Results and Discussions

The main contents of rice husk and residues are shown in Table 1. It is seen that the content of lignin, cellulose, and silica increased in residue 1 and residue 2, with the decrement of semicellulose.

### 3.1. Discussion on the Ethanediol Pulping of RH Residue 1

The mechanism of organosolv pulping is commonly considered as follows [19]: the *α*-aryl ether bond and *β*-aryl ether bond of lignin are attacked by the organic solution under the conditions of sufficient temperature, pressure, and time. The hydrolysis of *α*-aryl ether bond is the key factor affecting the organosolv pulping, but there are other reactions that occurred at the same time, such as the hydrolysis of *β*-aryl ether bond. The lignin matrix is broken and converted to the lignin fragment and then dissolved in the organic solution to form a stable colloidal dispersion. When the water is added in this colloidal dispersion, the concentration of organic solution is decreased; also the dissolvability of lignin fragment is decreased. So the lignin fragment is deposited and separated from the organic solution.

Therefore, the variables affecting the yield of lignin from residue 1 are considered as temperature, time, concentration of ethanediol solution, and the ratio of residue to ethanediol. The method of one factor experiments is carried out for investigating the effects of those variables on the yield of lignin in the pulping process. The yield of lignin in this paper is defined as (actual output of lignin)/(theoretical output of lignin) × 100.

### 3.2. The Influence of Pulping Temperature on the Yield of Lignin

**Figure 1:**
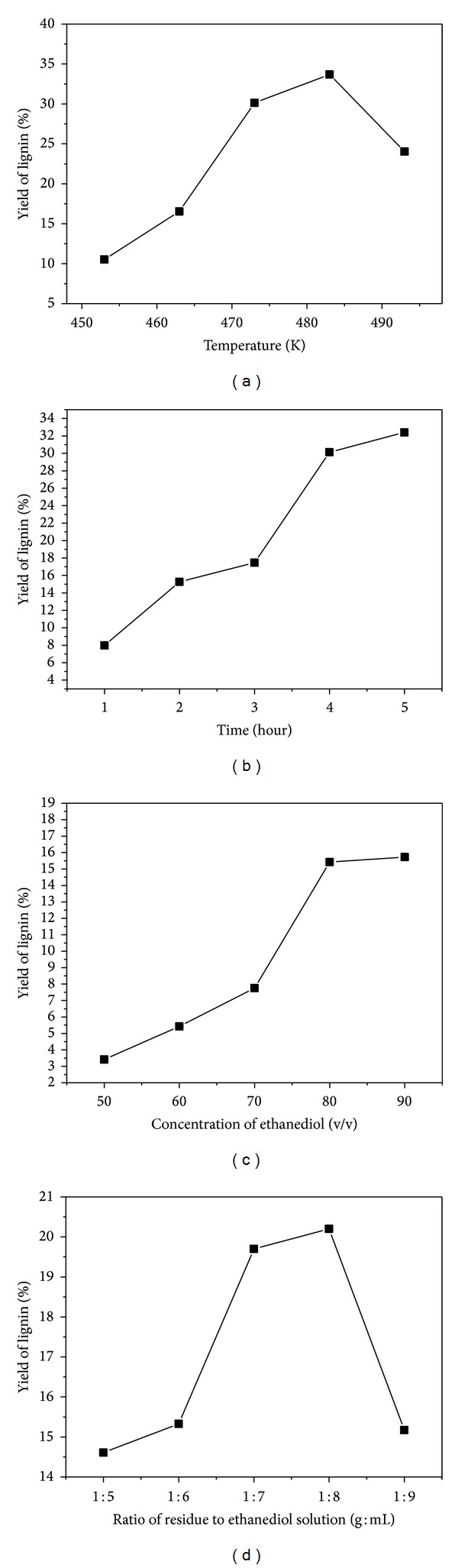
Effects of (a) temperature; (b) time; (c) concentration of ethanediol; and (d) ratio on the yields of lignin.

The influence of pulping temperature on the yield of lignin is investigated, that is, 453 K, 463 K, 473 K, 483 K, and 493 K. Autoclaves were nonisothermally heated to certain temperature and kept for 3 hours, respectively. The concentration of ethanediol was fixed at 80% (v/v); the RH residue 1 and ethanediol solution in fixed ratio (1 : 6, g : mL) were mixed in stainless steel autoclaves. The lignin samples were obtained after filtration and vacuum-drying at room temperature. The effects of temperature on the yield of lignin are shown in Figure 1(a).

It is seen from Figure 1(a) that the yield of lignin increased rapidly with the increment of temperature from 453 K to 483 K, but when the temperature exceeded 483 K, the yield of lignin fell off. It can be explained as the hydrolysis of lignin needing enough energy, but when the temperature exceeded 483 K, the side reaction of lignin increased rapidly.

### 3.3. The Influence of Pulping Time on the Yield of Lignin

The effect of time, that is, 1, 2, 3, 4, and 5 hours, was investigated. The concentration of ethanediol solution and temperature were fixed at 80% and 483 K. Residue 1 and ethanediol solution were mixed in fixed ratio of 1 : 6 (g : mL) in the stainless steel autoclaves. Those autoclaves are nonisothermally heated to 483 K and, respectively, kept for a certain time. After filtration and vacuum-drying at room temperature, lignin samples were obtained. The effects of pulping time on the yield of lignin are shown in Figure 1(b).

Figure 1(b) shows a remarkable increment of the lignin's yield with the increment of time before 5 hours. The delignification of biomass in organic solution was modeled as the irreversible and consecutive dissolution of initial, bulk, and residual lignin [20]. As we can see from Figure 1(b), the duration of 2 hours might be the initial dissolution of lignin, 3-4 hours the bulk, and 5 hours the residual dissolution of lignin.

### 3.4. Influence of Concentration of Ethanediol Solution on the Yield of Lignin

A series of ethanediol solutions with the concentration of 50%, 60%, 70%, 80%, and 90% (volume fraction) were, respectively, investigated. Then the autoclaves were nonisothermally heated to 483 K and kept for a further four hours. After being filtrated and vacuum-dried at room temperature, lignin samples were obtained. The influence of concentration on the yield of lignin is shown in Figure 1(c).

It is seen from Figure 1(c) that the yield of lignin increased rapidly with the increment of concentration before 80%, but when the concentration exceeds 80%, the yield of lignin changes little.

### 3.5. Influence of Ratio of Residue 1 to Ethanediol Solution on the Yield of Lignin

The effect of the ratio of residue 1 to ethanediol solution, 1 : 5, 1 : 6, 1 : 7, 1 : 8, and 1 : 9, (g : mL) was, respectively, investigated. The concentration of ethanediol aqueous solution is fixed at 80%, and the autoclaves were nonisothermally heated to 483 K and kept for a further four hours. The influence of the ratio of residue 1 to ethanediol solution on the yield of lignin is shown in Figure 1(d).

As Figure 1(d) shows, the ratio of residue 1 to ethanediol solution has no remarkable influence on the yield of lignin. The lowest and highest yield are 14.61% and 20.20%, respectively. This phenomenon may imply that the ethanediol solution is always enough for the dissolution of lignin.

The influence of pulping temperature, time, concentration of ethanediol, and the ratio of residue 1 to solution on the yield of lignin was discussed by the one-factor experiments here. However, those experiments cannot give the best combination of four factors or the key variable on the yield of lignin. So an orthogonal test design is carried out to optimize the lignin extraction; the details are shown as follows.

### 3.6. The Optimization of Ethanediol Pulping via the Orthogonal Experimental Design

The levels of each factor in orthogonal experimental are selected based on the one-factor experimental results. For example, the levels of temperature are chosen as 473, 483, and 493 K, for the one-factor experiments of temperature demonstrate that the optimal temperature is 483 K, so the value around 483 K is chosen for the orthogonal test. Independent variables with four varying levels, A (ethanediol concentration), B (ratio of residue 1 to ethanediol), C (time), and D (temperature), are listed in Table 2.

All the selected factors are examined using a L_9_(3)^4^ orthogonal test design. The total evaluation index is analyzed by statistical method. The analysis results performed by statistical software SPSS 13.0 are presented in Table 3.

According to the R value, the factors affecting the yield (%) of lignin are listed in a decreasing order as follows: D > A > C > B. The key factor influencing the yield is temperature. The ethanediol's concentration has a remarkable influence on the yield of lignin, but the ratio of residue 1 to the ethanediol has little effect, which agrees with the results of one-factor experiments. The maximum yield (34.47%) of the lignin is obtained when the temperature, ethanediol concentration, time, and ratio of residue 1 to ethanediol are 493 K, 90%, 4 h, and 1 : 6, respectively.

Although the optimal condition is discussed by orthogonal test, the highest lignin's yield is only 34.47%. The reason for this condition is due to the lack of semicellulose in the RH residue 1, for the lignin matrix is broken by the acetic acid generated from the acetyl group of semicellulose [20].

Therefore, the experiments of the catalyst's influence on the yield of lignin are carried out. The sulfuric acid is chosen to provide the H^+^ for its thermostability and less volatility.

### 3.7. Influence of Catalyst on the Yield of Lignin

**Figure 2:**
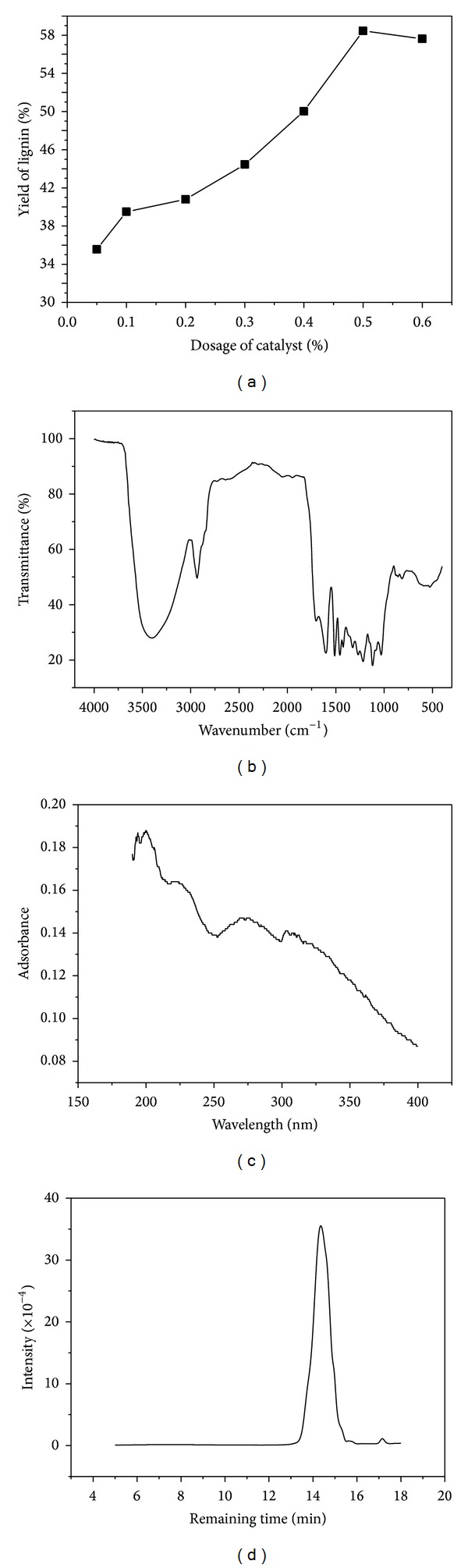
Effects of catalyst on the yield of lignin (a) and analysis of lignin: IR (b); UV (c); GPC (d).

The optimal conditions of orthogonal test are adopted, that is, 473 K, 4 hours, 1 : 6, and 90% in the catalytic experiments. The concentrated sulfuric acid is added in the mixer of residue 1 and ethanediol solution before the pulping. The dosage of catalyst is counted by the volume percentage of ethanediol solution.  Figure 2(a) shows the effects of catalyst on the yield of lignin.

It is seen from Figure 2(a) that sulfuric acid has a remarkable influence on the yield of lignin. The lignin's yield increased rapidly with the increment of catalyst's dosage. When the dosage of catalyst is 0.5%, the yield of lignin reached the highest value of 58.45%, which increased 70% more than the optimal result of orthogonal test. However, when the dosage of catalyst exceeded 0.5%, the lignin's yield dropped due to the subsidiary reaction. The experiment showed the residue had slight carbonization at the same time.

The lignin obtained from this optimal condition is investigated by the infrared spectrometer (IR), ultraviolet spectrometer (UV), and Gel Permeation Chromatography (GPC).

The IR spectrograph and explanation are shown in Figure 2(b) and Table 4, which show the characteristic absorption peak of lignin, such as 820 cm^−1^, 1201 cm^−1,^ and 1275 cm^−1^. This result indicates the lignin structure of the sample.

The UV spectrograph is shown in Figure 2(c), which also confirms the character structure of lignin. The strongest peak near 210 nm is an absorption band of the conjugative bond of alkenes, indicating *n* → π* electron transition and stronger undersaturation. The broad acromion that appeared around 300 nm is caused by the existence of the conjugated system such as aromatic ring.

GPC analysis is performed to determine Mw, Mn, and DPI of the lignin. As Figure 2(d) shows, the elution time shifted towards higher molecular weights, and the GPC trace remained monomodal. The Mw and Mn are 1527 and 1208, respectively. The polydispersity of the polymers is 1.26.

Through the catalyzed ethanediol pulping, the optimal conditions of lignin separation from residue 1 were investigated. The highest yield of lignin reaches 58.45%. The residue produced in the optimal catalyzed ethanediol pulping is named residue 2. It has more silica and less lignin than residue 1 (as shown in Table 1), which means the decrement of lignin waste and energy consumption in the pyrolysis progress. At the same time, most of metal elements in RH (such as K, Na, Ca, and Al) decreased dramatically after the semicellulose hydrolysis and lignin separation (see Table 5). So residue 2 is better than residue 1 to prepare the ultrafine silica. The experiments are carried out as follows.

### 3.8. Preparing Amorphous Ultrafine Silica from Residues via Hydrolysis

Variables such as temperature, atmosphere, and time have pronounced effects on the morphology and dispersity of ultrafine silica via pyrolysis of residue 2. The details are discussed as follows.

### 3.9. The Temperature of Pyrolysis

Temperature has important impact on the purity and morphology of ultrafine silica. Therefore, the TG analysis was employed to investigate the pyrolysis temperature. The results of rice husk, residue 1, and residue 2 were shown in Figure 4(a).

Figure 4(a) shows that the temperatures of constant weight for three samples increased with the increment of the ash contents, that is, 560°C for rice husk, 630°C for residue 1, and 680°C for residue 2. The reason for this difference is due to the decrease of semicellulose in residues. The thermostability of semicellulose, lignin, and cellulose is progressively increasing. Rice husk has the most abundant semicellulose; residue 1 has the most abundant lignin; residue 2 has the most abundant cellulose. Therefore, the rice husk has the lowest constant temperature for the most abundant semicellulose, and residue 2 has the highest constant temperature for the most abundant cellulose.

### 3.10. The Atmosphere and Time of Pyrolysis

The atmosphere and time have obvious effect on the morphology and the disparity of ultrafine silica. Experiments demonstrated that the direct hydrolysis of residue 1 in air atmosphere needs harsh conditions (750°C for 15 minutes or 600°C for six hours); the ultrafine silica had obvious agglomeration, too [18]. It is found that oxygen atmosphere can decrease the pyrolysis temperature and time comparing to the air atmosphere (600°C for 1 hour, 550°C for 1.5 hours), but the ultrafine silica still keeps the obvious agglomeration. The results of TG indicated that residue 2 is more difficult to pyrolyze for its high content of cellulose. Therefore, an indirect pyrolysis method is adopted to decrease the temperature of pyrolysis of residue 2, for the sake of improving the agglomeration of silica. This method includes two steps; therefore it is called the* two-step pyrolysis method *in this paper.

**Figure 3:**
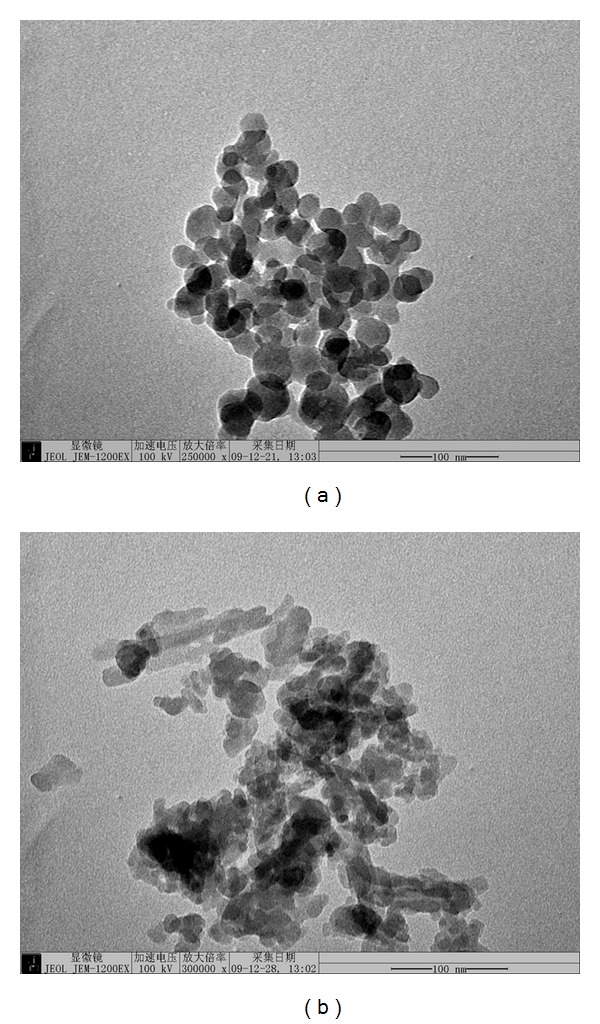
TEM of silica and carbon mixer (a) and ultrafine silica (b).

*(1) Pyrolyzing under Inert Atmosphere (The First Step)*. The dried residue 2 was weighted and put into a tube furnace, which was heated to 550°C beforehand. Residue 2 was pyrolyzed under the flowing of carbon dioxide gas for half an hour. For the inert characters of carbon dioxide, there was not the kindle of residue 2, which avoided the remarkable increment of temperature. The organic materials such as cellulose were hydrolyzed to the carbon, which kept the silica away from agglomeration. After the first step, the mixer of carbon and silica was obtained as Figure 3(a) shows.

*(2) Pyrolyzing under Oxygen Atmosphere (The Second Step)*. The carbon dioxide gas flow is changed to the oxygen flow under 550°C. The black powder completely turns into white silica in 30 minutes, with the carbon being pyrolyzed to carbon dioxide.

Therefore, the temperature of pyrolyzing residue 2 is decreased to 550°C by the two-step pyrolysis method, which is lower than the temperature of pyrolysis in air (680°C). It is helpful to improve the agglomeration of super fine silica powder.

The morphology, size, and aggregation of this silica powder are investigated by TEM. As Figure 3(b) shows, the powder is a kind of irregular aggregate with a diameter of 20 nm.

**Figure 4:**
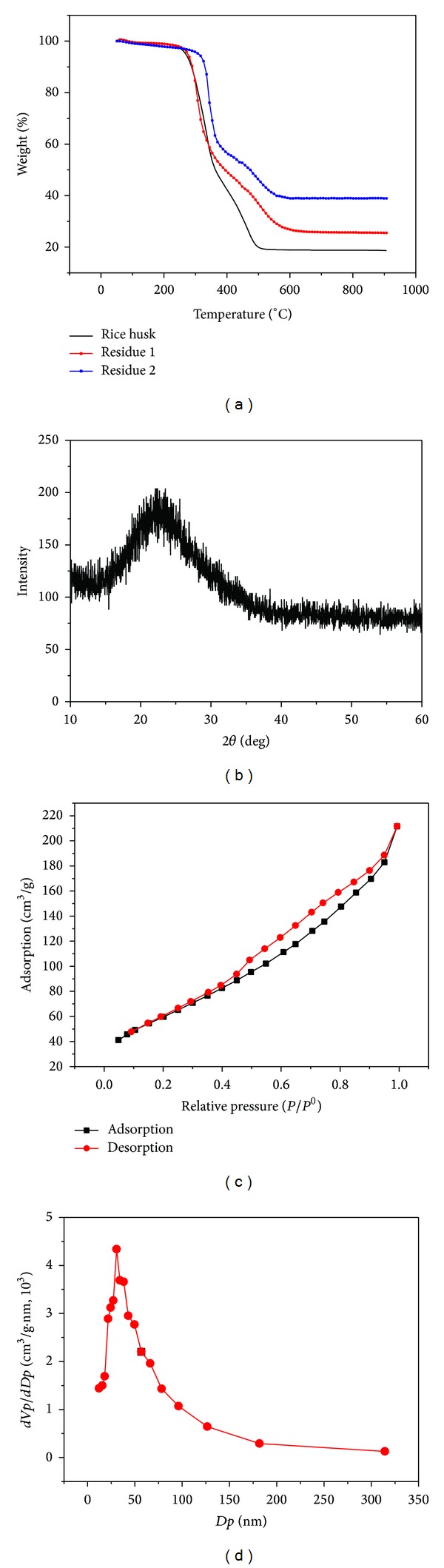
TG of rice husk and residues (a) and analysis of ultrafine silica; XRD (b); adsorption-desorption isotherms (c); differential pore size distribution (d).

The crystalline state of silica powder is tested by XRD, as Figure 4(b) shows. The result shows that the silica powder is amorphous state. The silica powder is porous; the Brunauer-Emmett-Teller (BET) method is used to analyze the porosity and specific surface area of the sample.  Figure 4(c) shows the nitrogen adsorption-desorption isotherms of silica powder at 77 K. The lower portion of the loop is traced out on adsorption, and the upper portion on desorption. It is seen that the hysteresis loop resembles type IV of Brunaur's classification. At low values of P/P^0^, the isotherm is similar to type II, but then adsorption increases markedly at P/P^0^ above 0.5, where pore condensation takes place.

Figure 4(d) shows a plot of the increment of pore volume per increment in pore size, versus pore size. This result is determined by the Barrett-Joyner-Halenda (BJH) method (based on the desorption branch). A single peak is observed in the silica samples having maxima at 30.6 nm. The pores may result from the residual spaces between the fine particles existing or formed in the silica preparation. The BET result tells the specific surface area of this silica is 225.20 m^2^/g. The reason for porosity is that the organic matter has been broken up during the thermal decomposition of rice husk, thus leaving a highly porous structure.

The purity for silica obtained from the rice husk and residue 2 under the optimum conditions of ethanediol pulping is investigated by ICP. The results are shown in Table 5. It is seen that the metal oxides in RH are removed efficiently after semicellulose hydrolysis and ethanediol pulping. The purity of silica is 99.92%, which is higher than the silica obtained from residue 1 via pyrolyzing in air atmosphere [18], with better dispersity and lower temperature of residue pyrolyzing.

## 4. Conclusions

Through sulfuric acid hydrolysis, ethanediol solution pulping, and the two-step pyrolysis, the high purity D-xylose, organosolv lignin, and ultrafine silica are prepared consecutively from rice husk, so the semicellulose, lignin, and silica in rice husk are comprehensively utilized. The optimal conditions for extracting lignin from residue 1 are 493 K, 90% (v/v), 4 h, and 1 : 6 (g : mL), with the maximum yield of 34.47%. When the dosage of catalyst (concentrated sulfuric acid) is 0.5%, the yield of lignin reached the highest 58.45%. Amorphous ultrafine silica is prepared from residual 2 via two-step pyrolysis method with the diameter around 20 nm, the specific surface of 225.20 m^2^/g, and the purity of 99.92% (wt.%).

## Figures and Tables

**Table 1:** The main contents of the rice husk, residue 1, and residue 2.

Content (wt.%)	Semicellulose	Lignin	Cellulose	Silica	Others
Rice husk	18.20	24.52	35.86	18.85	2.57
Residue 1	2.42	30.23	40.61	25.48	1.26
Residue 2	—	4.44	56.09	38.91	0.57

“—” represents the content was undetected.

**Table 2:** Factors and levels for L_9_(3)^4^ orthogonal test.

Variable	Levels		
	1	2	3
Ethanediol's concentration (v/v)	70	80	90
Ratio of residue to ethanediol (g : mL)	1 : 4	1 : 6	1 : 8
Time (hour)	4	5	6
Temperature (K)	473	483	493

**Table 3:** Analysis of L_9_(3)^4^ orthogonal test results.

Number	(*A*) Ethanediol concentration	(*B*) Ratio of residue 1 to ethanediol	(*C*) Time	(*D*) Temperature	The yield of lignin
1	1	1	1	1	10.64
2	1	2	2	2	16.01
3	1	3	3	3	24.01
4	2	1	2	3	26.78
5	2	2	3	1	18.86
6	2	3	1	2	28.27
7	3	1	3	2	33.60
8	3	2	1	3	34.47
9	3	3	2	1	18.09
K1	16.89	23.67	24.46	15.86	
K2	24.64	23.11	20.29	25.96	
K3	28.72	23.46	25.49	28.42	
*R*	11.83	0.56	5.20	12.56	

**Table 4:** Explanation of IR spectrograph.

Position/cm^−1^	Band origin
3400	–OH stretching
2943	C–H stretching
1706	C=O stretching nonconjugated to the aromatic ring
1610	C=O stretching conjugated to the aromatic ring
1520	Aromatic ring vibrations
1462	Aromatic ring vibrations C–H deformations
1275	Aromatic ring vibrations of guaiacyl
1201	C–O stretching of syringy
1124	Aromatic ester
1027	C–H and C–O deformation
820	Syringy C–H vibrations

**Table 5:** Contents of metal oxide in silica obtained from the rice husk and residue 2 under the optimum conditions of ethanediol pulping.

Metal oxide (wt.%)	K_2_O	Na_2_O	CaO	MgO	Al_2_O_3_	Fe_2_O_3_	CuO	MnO_2_	ZnO	Sum
From RH	0.9808	0.3689	7.9216	1.7877	0.4530	0.3158	0.0047	0.1121	0.1145	12.0616
From residue 2	0.0113	0.0145	0.0235	0.0011	0.0124	0.0035	0.0017	0.0000	0.0036	0.0716
